# Shape-Memory and Anisotropic Carbon Aerogel from Biomass and Graphene Oxide

**DOI:** 10.3390/molecules26185715

**Published:** 2021-09-21

**Authors:** Zilu Lin, Wenzhao Jiang, Zehong Chen, Linxin Zhong, Chuanfu Liu

**Affiliations:** State Key Laboratory of Pulp and Paper Engineering, South China University of Technology, Guangzhou 510641, China; 15813140671@163.com (Z.L.); jiangwenzhao@cvte.com (W.J.); chenzehong24@126.com (Z.C.)

**Keywords:** biomass, bioresource, carboxymethyl cellulose, aerogel, carbon

## Abstract

Biomass, as the most abundant and sustainable resource on the earth, has been regarded as an ideal carbon source to prepare various carbon materials. However, manufacturing shape-memory carbon aerogels with excellent compressibility and elasticity from biomass remains an open challenge. Herein, a cellulose-derived carbon aerogel with an anisotropic architecture is fabricated with the assistance of graphene oxide (GO) through a directional freeze-drying process and carbonization. The carbon aerogel displays excellent shape-memory performances, with high stress and height retentions of 93.6% and 95.5% after 1000 compression cycles, respectively. Moreover, the carbon aerogel can identify large ranges of compression strain (10–80%), and demonstrates excellent current stability during cyclic compression. The carbon aerogel can precisely capture a variety of biological signals in the human body, and thus can be used in wearable electronic devices.

## 1. Introduction

Carbon aerogels with a three-dimensional network have gained great attention in the applications in various wearable devices such as pressure sensors, flexible electrochemical energy storage, and others [1,2,3]. To meet these applications, carbon aerogels are required to possess shape-memory performances, such as reversible compressibility, elasticity, fatigue resistance, and high sensitivity. Recently, some carbon aerogels with compressibility and elasticity have been fabricated by several nano building blocks, such as carbon nanotubes (CNT) [4,5,6], graphene (including graphene oxide and GO) [7,8,9,10], transition metal carbide/nitride (MXene) [11,12], and their composites. For instance, Jiang et al. [13] prepared an MXene/reduced graphene oxide (rGO) aerogel with high compressibility and elasticity, which can be applied in piezo resistive sensors. However, the above-mentioned nano materials are expensive, and their synthesis process will cause environmental issues, thus limiting their industrial application. Reducing the cost and environmental pollution is essential to the development of carbon aerogels.

Biomass, as the most abundant and sustainable resource on the earth, has been regarded as an ideal carbon source to prepare various carbon materials. A series of carbon aerogels based on biomass resources such as chitosan [14], kapok [15], waste paper [16], poplar catkin [17], bacterial cellulose [18], and wax gourds [18] have been prepared through freeze-drying and subsequent carbonization. Nonetheless, manufacturing shape-memory carbon aerogels with excellent compressibility and elasticity from biomass remains an open challenge. The disordered three-dimensional texture causes structural damage at a high compression strain, resulting in poor shape-memory performance. The reasonable design of a microstructure that facilitates the effective transfer of stress and recovers its shape under high compression strain is highly required. Anisotropic, yet ordered, structure is ubiquitous in nature and exhibits high mechanical strength, fatigue resistance, and efficient ion conducting or mass transport at specific direction, such as wood and bamboo [19,20,21,22,23].

Here, a shape-memory and anisotropic cellulose-derived carbon aerogel is fabricated with the assistance of graphene oxide (GO) through a directional freeze-drying process followed by carbonization. The reduction in the usage of GO will significantly lower the cost of carbon aerogel and pollution. This anisotropic structure composed of wavy carbon lamellas of the carbon aerogel enables the efficient transfer of external stress along the whole structure, thus allowing an excellent shape-memory performance at high compression strain. Moreover, the carbon aerogel can precisely capture a variety of biological signals of human body, indicating its potential application in wearable electronic devices.

## 2. Results and Discussion

GO was prepared via a modified Hummers method [24,25,26]. GO presents a gauze-like shape with fewer layers, which indicates the good peeling effect of GO nanosheets (Figure S1). The FTIR spectrum of the prepared GO (Figure S2) shows a broad absorption band at 3250 cm^−1^, which can be assigned to the stretching vibration of O-H on GO nanosheets [27,28]. The peak at 1725 cm^−1^ is caused by the stretching vibration of C = O in carbonyl and carboxyl groups at the edge of GO nanosheets. The absorption peak at 1621 cm^−1^ is attributed to the O-H bending vibration of water molecules. The peak at 1048 cm^−1^ is the vibration absorption peak of C-O-C. The results indicate that graphite was successfully oxidized. In addition, the presence of the as mentioned polar groups makes the surface of GO nanosheets negatively charged after ultrasonic treatment in the polar solvent (such as water solution, etc.). Therefore, stable GO dispersion can be formed by electrostatic forces.

**Figure 1:**
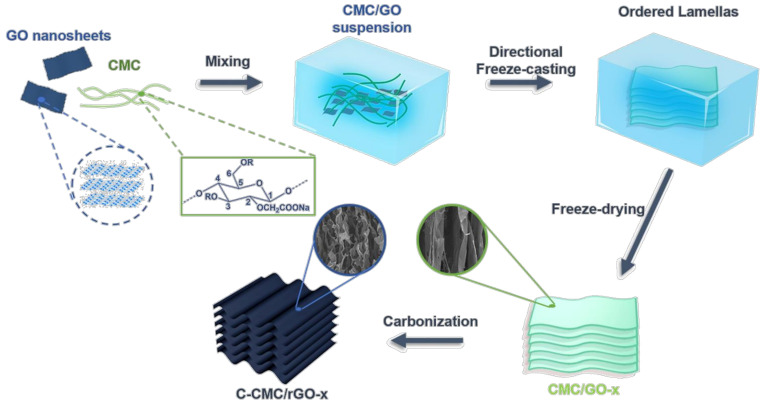
The fabrication process of carbon aerogel C-CMC/rGO-x.

**Figure 2:**
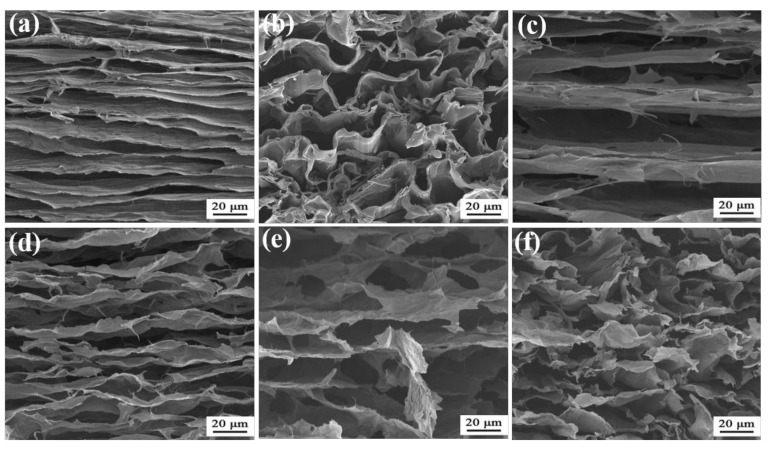
SEM images of (**a**) A-CMC, (**b**) C-CMC, (**c**) A-CMC/GO-4, (**d**) C-CMC/rGO-4, (**e**) C-CMC/rGO-1, and (**f**) C-CMC/rGO-8.

As shown in Figure 1, by mixing carboxymethyl cellulose (CMC) and GO nanosheets in water, directional freezing, freeze drying and annealing, carbon aerogel could be fabricated. Directional freezing and the assistance of GO nanosheets are critical factors in creating an anisotropic and ordered structure with wavy lamellas. During directional freezing, the ice grows from one end (with liquid nitrogen) to the other end, extruding CMC to form an anisotropic and lamellar texture (Figure 2a). After carbonization, the lamellas curl seriously and become disordered, which is caused by the serious shrinkage at high temperature (Figure 2b). With GO nanosheets, however, the aerogel exhibits anisotropic and lamellar texture (Figure 2c). The lamellas are well preserved and wavy after annealing (Figure 2d), demonstrating that GO nanosheets can prevent the structure from serious shrinkage.

As shown in Figure S3, after carbonization, pure CMC aerogel deforms and shows a significant volume shrinkage; while CMC/GO aerogel can retain its integration and show no obvious volume shrinkage, indicating that the presence of GO helps to maintain the microstructure of aerogel at a high temperature. Figure S4 shows the FTIR spectra of A-CMC, A-CMC/rGO-4 (1.4 wt%) and C-CMC/GO-4. The peaks in the spectrum of A-CMC appearing at 3355, 1590, 1414, 1322, and 1056 cm^−1^ can be assigned to the stretching vibration of -OH (hydroxyl) groups, C-O (carboxyl), C-H_2_ wagging modes, and the in plane bending of -OH and C-O (alkoxy) groups, respectively. In addition, a new peak at 1725 cm^−1^ corresponding to the stretching of C = O (carboxyl and carbonyl) on GO can be observed in the spectrum of A-CMC/rGO-4 (1.4 wt%), indicating the combination of GO and CMC. Meanwhile, after the cooperation of GO, the adsorption peak of -OH at 3355 cm^−1^ (A-CMC) generates bathochromic shift (3438 cm^−1^ for A-CMC/GO-4), suggesting the hydrogen bonding between CMC and GO. After carbonization, most of the peaks corresponding to the oxygen-containing groups disappear and become sparse in the spectrum of C-CMC/rGO-4, demonstrating that most of the oxygen functional groups of CMC and GO have been removed during carbonization. Moreover, two peaks appear at 1626 and 1114 cm^−1^, which are attributed to the C = C of carbon skeleton (benzene ring structure) and the C-H bending vibration of benzene ring in plane, respectively, implying that CMC has been converted into carbon material after carbonization.

To find out the influence of GO content on the structure and mechanical properties of material (solid content of 2.8 wt%), C-CMC/rGO-x with different weight ratios of CMC to GO were prepared. As shown in Figure S5, with the increase of the GO ratio, the volume shrinkage of carbon aerogel reduces, indicating that the increasing GO content is beneficial to enhance the thermal stability of aerogels. Among these carbon aerogels, C-CMC/rGO-4 with mass ratio of 4:1 shows better morphology, while C-CMC/rGO-1 (mass ratio of CMC to GO = 1:1) with the highest GO content reveals significant cracks on the surface. C-CMC/rGO-1 consists of mussy and discrete fragments with a large space between these fragments (Figure 2e), indicating that the amount of carbon derived from CMC is insufficient to connect GO nanosheets into successive sheets at low CMC content, resulting in discontinuous lamellar structure. When GO content is low, the carbon aerogel C-CMC/rGO-8 also displays disordered and discrete structures (Figure 2f), suggesting that only with enough GO nanosheets the continuous layers can be formed. Hence, an appropriate GO content promotes the formation of highly ordered lamellar structure by inducing the generation of parallel lamellas.

**Figure 3:**
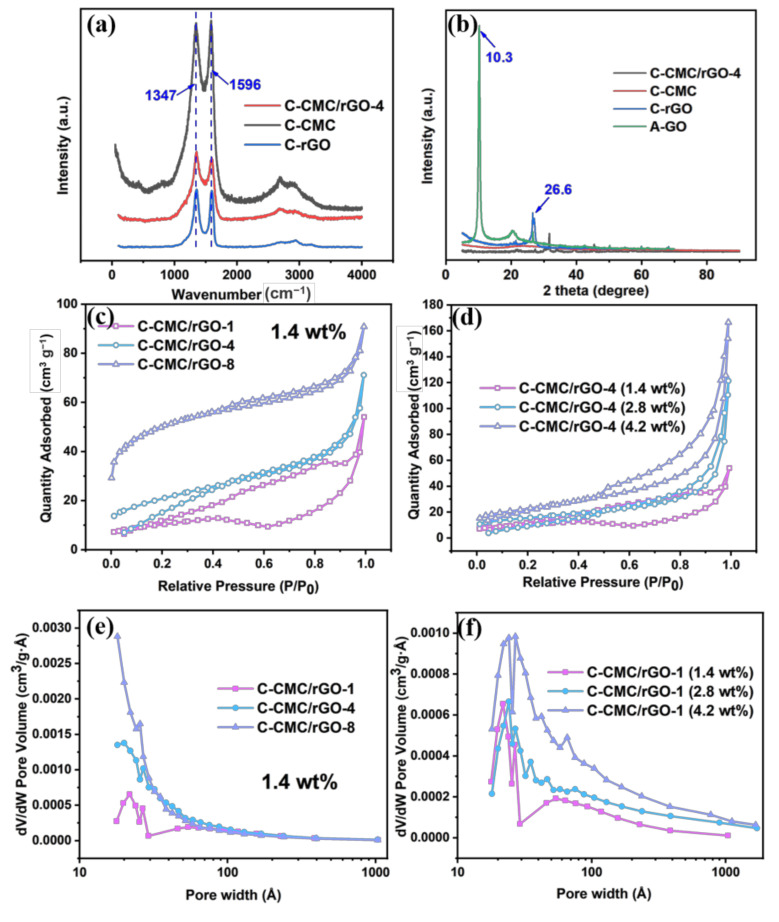
(**a**) Raman patterns of C-CMC, C-CMC/rGO-4 and C-rGO. (**b**) XRD patterns of C-CMC/rGO-4, C-CMC, C-rGO, and A-GO. The N_2_ adsorption-desorption isotherms of (**c**) C-CMC/rGO-1, C-CMC/rGO-4 and C-CMC/rGO-8 (1.4 wt%), and (**d**) C-CMC/rGO-1 (1.4 wt%), C-CMC/rGO-1 (2.8 wt%) and C-CMC/rGO-1 (4.2 wt%). The pore size distribution of (**e**) C-CMC/rGO-1, C-CMC/rGO-4 and C-CMC/rGO-8 (1.4 wt%), and (**f**) C-CMC/rGO-1 (1.4 wt%), C-CMC/rGO-1 (2.8 wt%) and C-CMC/rGO-1 (4.2 wt%).

As shown in Figure 3a, at 1347 cm^−1^ (D band) and 1596 cm^−1^ (G band), the reduced graphene oxide carbon aerogel (C-rGO) annealed at 750 °C shows two significant peaks. The D band is generated by the vibration of sp^2^ hybridized carbon, indicating disordered carbon structure or defect. G band is caused by the stretching of sp^2^ hybridized atom pair in carbon rings, which reflects the symmetry and order of carbon. The intensity ratio of the D band to G band (I_D_/I_G_) reflects the graphitized degree in carbon [29]. The I_D_/I_G_ value of C-rGO is 1.02, demonstrating the partial graphitization of C-rGO. C-CMC and C-CMC/rGO-4 also shows two peaks at D band and G band, with I_D_/I_G_ values of 0.85 and 1.08. In comparison to C-rGO, the relatively higher I_D_/I_G_ value of C-CMC /rGO-4 represents the increase of carbon defect from CMC-derived carbon.

The N_2_ adsorption-desorption isotherms of the carbon aerogels are shown in Figure 3c,d, and the corresponding specific surface area (SSA) and pore volume are summarized in Table S1. The SSA of the carbon aerogels with a 1.4 wt% solid concentration increases with the rise of the CMC content (i.e., 35.7 m^2^/g for C-CMC/rGO-1, 72.9 m^2^/g for C-CMC/rGO-4, and 179.3 m^2^/g for C-CMC/rGO-8), which can be attributed to the increasing micropores and mesopores, as indicated by the pore size distribution (PSD) in Figure 3e. Thus, it can be suggested that the conversion of CMC into carbon during pyrolysis can lead to more nanopores. Meanwhile, as the solid concentration rises, the carbon aerogels also reveal an enhanced SSA (Table S1), and display a combination of micropores and mesopores with a peak at about 2.4 nm (Figure 3f).

The XRD patterns of carbon aerogels in Figure 3b show that A-GO exhibits a sharp peak at 2*θ* = 10.3°, which can be attributed to GO (100) (Figure S6) [30]. During the oxidation in GO preparation, a great number of functional groups are embedded in graphite layers, which changes the inherent structure of the graphite, resulting in a lattice deformation. After the annealing treatment, a new peak at *2θ* = 26.6° representing the typical graphitic plane (002) [31,32,33] can be observed in the XRD spectrum of C-rGO, while the peak at 2*θ* = 10.3° disappears. C-CMC shows no peak, indicating an amorphous structure with disordered carbon.

The TGA curves of samples are shown in Figure S7. CMC exhibits a high mass retention of 41% at 700 °C, which is much higher than those of cellulose (A-CEL) and hydroxypropyl methyl cellulose (A-HPMC). The high weight retention of CMC is beneficial to prevent aerogel from serious volume shrinkage at high temperatures. GO has a mass retention at 36% at 700 °C [34,35], which fulfills the range of weight retention of GO (20%–50%) at 700 °C [32,33]. Therefore, A-CMC/GO-4 has a relatively high weight retention (33.6%) at 700 °C. During annealing, the large quantity of oxygen-containing groups on GO are eliminated and the van der Waals force forms between the layers of hexagonal carbon basal planes [35].

Figure S8 shows the mechanical performances (including compressibility, elasticity, and cyclic compression) of the as-prepared aerogels and carbon aerogels. When being compressed, CMC aerogel (A-CMC) without carbonization easily deforms, and the height reduces to 50.3% of its original height for only one cycle, exhibiting poor elasticity (Figure S8a). With the addition of GO, however, CMC/GO aerogel (A-CMC/GO) can undergo 50% compression strain for 100 cycles and remains a high stress retention (80.4%, Figure S8b). Therefore, GO can significantly enhance the mechanical performances of aerogel.

**Figure 4:**
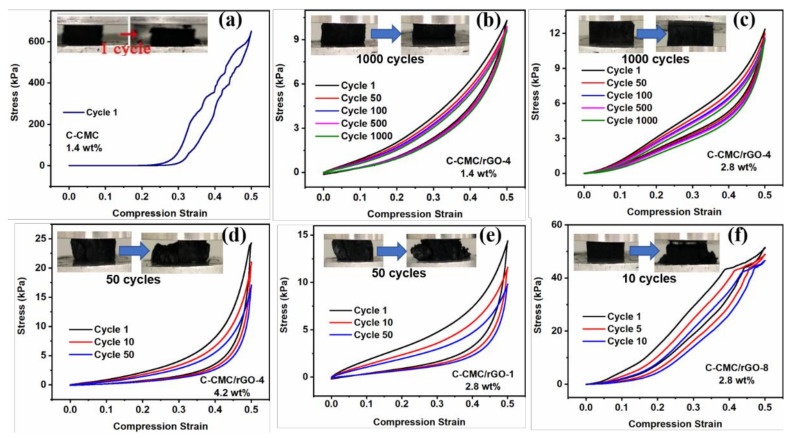
Stress-strain curves of (**a**) C-CMC, (**b**) C-CMC/rGO-4 with solid contents of 1.4%, (**c**) 2.8%, and (**d**) 4.2%. Stress-strain curves of (**e**) C-CMC/rGO-1 and (**f**) C-CMC/rGO-8.

Figure 4 demonstrates the mechanical properties of C-CMC/rGO-x. Carbon aerogel C-CMC is fragile and tends to collapse for only one cycle at 50% strain (Figure 4a). However, CMC/GO carbon aerogel (C-CMC/rGO-4) can withstand 1000 cycles at 50% compression strain and maintain its macrostructure and no obvious plastic deformation is observed (Figure 4b). The stress retention and height retention of C-CMC/rGO-4 (1.4% solid content) are high up to 93.6% and 95.5%, respectively, which is a sharp contrast to C-CMC. The result demonstrates that GO also remarkably improves the mechanical strength of carbon aerogel, which is likely due to the well-maintained structure and the intrinsic high strength of GO. Furthermore, the stress strain hysteresis ring in the curve is small, indicating no significant plastic deformation during cyclic compression owing to the reversible compression.

Figure S9 shows the stress retentions of C-CMC/rGO-4 with different solid contents at 50% strain. The C-CMC/rGO-4 (1.4 wt%) possesses the highest stress retention rates after 50, 100, 500 and 1000 cycles, at 97%, 95,3%, 94.7% and 93.6% respectively, while the stress retention of C-CMC/rGO-4 (4.2 wt%) with 50 cycles is significantly low at 68.3% and the aerogel was deformed severely under the press. The stress retentions of C-CMC/rGO-4 (2.8 wt%) are slightly lower than the stress retentions of C-CMC/rGO-4 (1.4 wt%) at 96.8% (50th cycle), 93.5% (100th cycle), 92.7% (500th cycle) and 92.2% (1000th cycle).

Figure S10 shows the stress curves of C-CMC/rGO-4 (1.4% solid content) after undergoing 80% strain for 100 cycles from three directions, e.g., the top, lateral and front, respectively. Compared to the high stress retention rate (93.6%) by pressing the aerogel from top, the stress retention rate obtained from lateral and front compression are significantly low, 70.9% and 15.6%, respectively. The results indicate the anisotropic structure of the carbon aerogel. The resistances of the aerogels in three directions (top, lateral and front) were 44.10 mΩ·cm, 19.36 mΩ·cm, and 15.80 mΩ·cm, respectively, also demonstrating the anisotropic structure.

The stress retention (92.2%, 1000 cycles, 50% strain) of C-CMC/rGO-4 with 2.8% solid content is almost the same to that of carbon aerogel with 1.4% solid content, but the stress (12.4 kPa) is higher than that of C-CMC/rGO-4 with 1.4% solid content (10.1 kPa). However, when the solid content increases to 4.2%, severe structural collapse happens to the carbon aerogel after 50 cycles at 50% strain, with a low stress retention of 68.3%. As shown in Figure S11, at 4.2% solid content, the microstructure of the carbon aerogel is plain, thick, and brittle, and thus the stress cannot efficiently transfer throughout the microstructures and fail to recover its shape.

At a high GO content, C-CMC/rGO-1 tends to collapse after 50 cycles, with a low height retention (Figure 4e). However, low GO content (i.e., 8:1) also results in the collapse of carbon aerogel (C-CMC/rGO-8, Figure 4f), which may be attributed to its random microstructures (Figure 2f). The disordered or inconsecutive fragments with many defects will easily cause stress concentration during compression, disabling the efficient transfer of stress within the three-dimensional network, finally resulting in the structural failure. On the contrary, the ordered and consecutive layers of C-CMC/rGO-4 (Figure 2d) allow the efficient transfer of stress along the entire network by extending the wavy microstructure, leading to a small deformation of the carbon layer. Upon the compression force release, the extended microstructure can recover to its original shape. Therefore, a reasonable mass ratio of CMC to GO is very important for the design of ordered, continuous, wavy layered structure to efficiently transfer stress and reversibly recover its shape.

**Figure 5:**
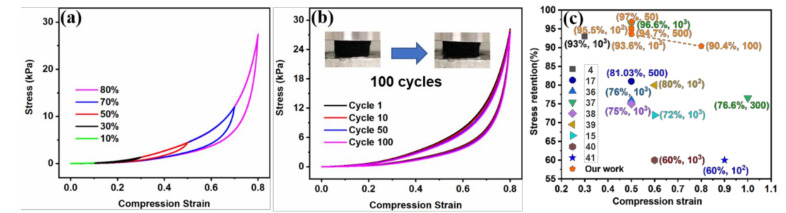
(**a**) Stress-strain curves of C-CMC/rGO-4 (1.4% solid content) at different compression strains. (**b**) stress-strain curves for 100 cycles at 80% strain. (**c**) the comparison of stress retention at different compression cycles of C-CMC/rGO-4 with those of other carbon aerogels (stress retentions at different compression cycles are shown in the parenthesis).

The stress curve of C-CMC/rGO-4 (1.4% solid content) steepens with strain from 10% to 80% (Figure 5a). The carbon aerogel not only withstands high compression deformation (80% strain) with 91.3% height retention after 100 compression cycles, but also displays 90.4% stress retention (Figure 5b), further demonstrating the excellent compressibility and ultra-high elasticity of C-CMC/rGO-4. Figure 5c and Table S2 indicate that the mechanical performances (at 50% and 80% strain) of C-CMC/rGO-4 are better than those of many other carbon aerogels reported previously, such as robust polyimide/carbon nanotube composite aerogel [4], poplars catkins carbon aerogel [17], graphene-based cellular monoliths [36], and so on [15,37,38,39,40,41].

**Figure 6:**
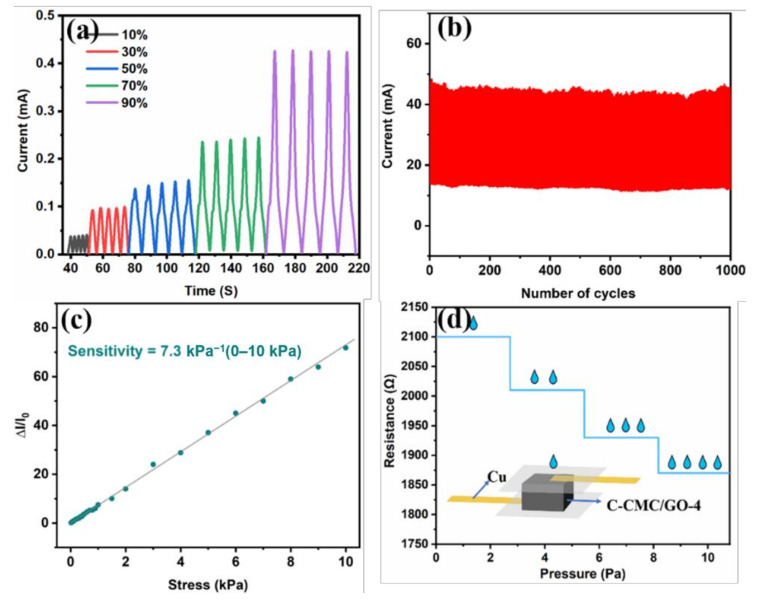
Current responses to strain and pressure and the linear sensitivity of carbon aerogel. (**a**) Current change caused by various compression strains. (**b**) The stability of current at 50% strain for 1000 cycles. (**c**) Linear sensitivity from 0 to 10 kPa. (**d**) Resistance as a function of water drops.

Figure 6 shows the electrical response behavior and the sensitivity of C-CMC/rGO-4. Figure 6a illustrates the responding currents at different strains of C-CMC/rGO-4. The current signal rapidly rises and decreases when strain is applied and released, demonstrating the reduced resistance when the compression strain increases because of the increasing contact area between the wavy lamellas. Furthermore, as the strain rises from 10% to 80%, the current rises significantly, illustrating that C-CMC/rGO-4 can precisely capture wide strain. It also exhibits excellent current stability during cyclic compression (Figure 6b).

Sensitivity (S) is defined as *S* = *δ*(Δ*I/I*_0_)/*δP*, where Δ*I* is the relative change of current, *I*_0_ is the initial current without pressure applied, and *δP* is the change of applied pressure. Figure 6c demonstrates the linear sensitivity of C-CMC/rGO-4 from 0 to 10 kPa (equivalent to 0–50% compression strain) is 7.3 kPa^−1^, illustrating a high linear sensitivity to detect a wide range of pressure. The pressure detection limit was measured by dropping water on the assembled sensor, and each water drop equates to 2.75 Pa applied on the sensor (Figure 6d). It is found that the detection limit is as low as 2.75 Pa, and the current increases with increasing number of water drops.

**Figure 7:**
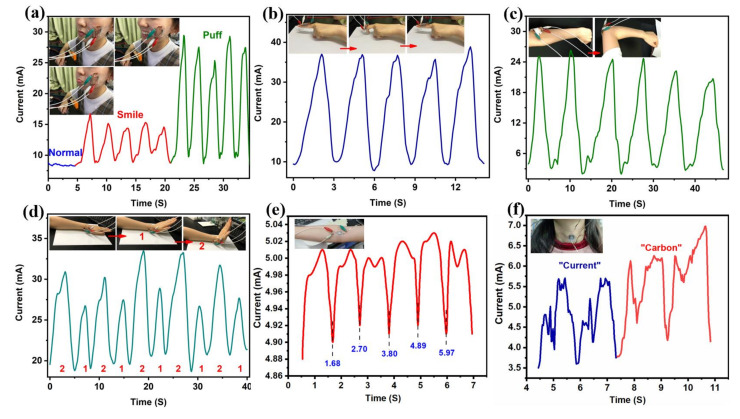
Application of C-CMC/rGO-4 in detecting biosignals from human. Current signals from (**a**) face expressions, (**b**) finger, (**c**) elbow, (**d**) wrist bending, (**e**) pulse and (**f**) vocal cords.

Considering its excellent fatigue resistance and high linear sensitivity, C-CMC/rGO-4 is assembled as a wearable sensor for detecting biosignals. Figure 7a exhibits that the as-prepared device can sense electrical signals from smiling or puffing. Furthermore, the sensor can monitor joint movements (such as fingers, elbows, and wrists) (Figure 7b,d). The current increases rapidly when the finger or elbow is bent (Figure 7b,c). By bending the wrist, repeatable signals can be obtained (Figure 7d). Apart from that, the sensor can monitor the pulse beat and the vibration of vocal cords (Figure 7e,f). Thus, the excellent mechanical performance and high linear sensitivity make C-CMC/rGO-4 a promising candidate in wearable and portable sensing electronic products.

## 3. Experiments and Methods

### 3.1. Materials

Carboxymethyl cellulose (CMC) was purchased from Macklin (Shanghai, China). Graphite powders were purchased from Nanjing XFNANO Materials Tech Co., Ltd (Nanjing, China). 98 wt% H_2_SO_4_ and KMnO_4_ were purchased from Shanghai Aladdin Biochemical Technology Co., Ltd. (Shanghai, China).

### 3.2. Preparation of CMC/GO Carbon Aerogel

#### 3.2.1. Preparation of CMC/GO Suspension

GO powder was prepared through oxidizing graphite powders via a modified Hummers method [24,25,26]. Firstly, the NaNO_3_ and H_2_SO_4_ solutions were carefully mixed in an ice bath. KMnO_4_ was added slowly in portions to keep the reaction temperature below 20 °C. The water was added at 35 °C and kept stirring for 30 min, producing a large exotherm to 98 °C. Additional water (420 mL) and 30% H_2_O_2_ (3 mL) were added, producing another exotherm. After air cooling, the mixture was centrifuged until the supernatant was neutral. The black residues were strained out, and then the obtained GO suspension was ultrasonicated (KQ-100B) for 10 min. To obtain the CMC/GO suspension, CMC powder was added into the as-prepared GO suspension (1.8 wt%). The above mixture was stirred for 12 h (500 rpm) and then ultrasonicated for another 1 h to remove air bubbles and ensure the homogeneous mixture of CMC and GO.

#### 3.2.2. Fabrication of Aerogels

A plastic box (38 mm × 29 mm × 22 mm) filled with CMC/GO suspension was placed on a steel plat contacting with liquid nitrogen to directionally freeze the CMC/GO mixture. After freeze drying for 60 h, CMC/GO aerogels, named as A-CMC/GO-x (x represents the mass ratio of CMC to GO, x = 1, 4, 8, i.e., 1:1, 4:1, 8:1, respectively) were obtained. Aerogels with solid contents of 1.4 wt%, 2.8 wt%, and 4.2 wt% were also attained by the same method. In addition, pure CMC aerogel (A-CMC) and pure GO aerogel (A-GO) were also fabricated from pure CMC solution and GO suspension, respectively.

#### 3.2.3. Preparation of Carbon Aerogels

The as-prepared aerogels mentioned above were carbonized in a tube furnace and experienced three stages under N_2_ atmosphere. The first stage was carried out from room temperature to 300 °C with a heating rate of 5 °C min^−1^. In the second stage, the annealing was performed from 300 °C to 400 °C with a heating rate of 0.5 °C min^−1^ and held at 400 °C for 1 h. Finally, aerogel was pyrolyzed to 750 °C (5 °C min^−1^) and held for 2 h. The obtained carbon aerogels were named as C-CMC/rGO-x, C-CMC, and C-rGO.

### 3.3. Characterizations

The microstructures of GO nanosheet, aerogels and carbon aerogels were observed using JEM-2100F transmission electron microscopy (JEOL, Akishima, Japan) and scanning electron microscopy (Zeiss, Oberkochen, Germany). X-ray diffraction (XRD) patterns of samples were recorded on a Bruker D8 diffractometer (Bruker, Mannheim, Germany). Thermal weight loss of samples were carried out on TG/DSC-200 analyzer (NETZSCH, Selb, Germany) in N_2_ atmosphere (10 °C min^−1^). Infrared and Raman spectra of samples were recorded on a VERTEX 70 Fourier transform Infrared spectrometer (Bruker, Mannheim, Germany) and LabRAM ARAMIS Raman spectrometer (HORIBA Jobin Yvon, Villeneuve d’Ascq, France), respectively. The electrical current at the compression and release cycle was measured using a CHI 660E electrochemical workstation (Chenhua Instrument Co., Ltd., Shanghai, China) The mechanical performances (including compression, elasticity, and fatigue resistance) of aerogel and carbon aerogels were performed on an Instron 5565 mechanical instrument (Instron, Norwood, MA, USA). The N_2_ adsorption-desorption measurement was operated at −196 °C to measure the specific surface area (SSA) and pore structure by a physisorption analyzer ASAP2460 (Micromeritics, Norcross, GA, USA). Before the N_2_ adsorption-desorption testing, all samples were degassed under vacuum at 180 °C for 10 h. The SSA was analyzed using the Brunauer-Emmett-Teller (BET) equation, while the pore size distribution (PSD) was calculated using the BJH method. The resistances from different directions were tested using an ST-2258C multifunction digital four-probe tester.

### 3.4. Assembly and Testing of Sensor

The C-CMC/rGO-4 based pressure sensor was prepared by assembling C-CMC/rGO-4 into two pieces of PET substrates with Cu wire. Strain or pressure were applied by an Instron 5565 mechanical instrument, while the current was recorded on a CHI 660E electrochemical workstation (with 1 V voltage).

## 4. Conclusions

We successfully fabricated a shape-memory and anisotropic carbon aerogel from carboxymethyl cellulose (CMC) with the assistance of graphene oxide through directional freeze drying and carbonization. The anisotropic and wavy lamellar structure endows excellent compressibility and elasticity to the carbon aerogel. The carbon aerogel can withstand 50% strain for at least 1000 cycles or 80% strain for at least 100 cycles, with high stress and height retention. It also exhibits rapid current response to strain or pressure, and a desirable linear sensitivity. Moreover, a wearable device can be assembled from the carbon aerogel for precisely capturing various signals caused by the movements of the human body. This work provides a solution for the preparation of hyper-elastic carbon aerogel from biomass resources.

## Data Availability

The data presented in this study are available on request from the corresponding author.

## Supplementary Materials

Figure S1: Transmission electron microscopy (TEM) image of GO. Figure S2: FTIR spectrum of GO. Figure S3: Digital photographs of aerogels before and after annealing. Figure S4: FTIR spectrum of C-CMC/rGO-4 and A-CMC/GO-4 (1.4 wt%). Figure S5: Digital photographs of C-CMC/rGO-x carbon aerogels with different mass ratios of CMC to GO. Figure S6: Raman patterns of A-CMC/rGO-4 and A-GO. Figure S7: TGA curves of A-CMC, A-GO, A-HPMC, A-CEL, and A-CMC/GO-4. Figure S8: Stress-strain curves of (a) A-CMC and (b) A-CMC/GO-4. Figure S9: Stress retentions of C-CMC/rGO-4 with different solid contents at 50% strain. Figure S10: Stress-strain curves of C-CMC/rGO-4 from (a) the front, (b) lateral and (c) the top. Figure S11: SEM image of C-CMC/rGO-4 with 4.2% solid content. Table S1: BET surface area of the carbon aerogels. Table S2: The comparison of stress retention of C-CMC/rGO-4 with those of other carbon aerogels.

## Sample Availability

Samples of the compounds are available from the authors.

## References

[1] Jian M., Xia K., Wang Q., Yin Z., Wang H., Wang C., Xie H., Zhang M., Zhang Y. (2017). Flexible and Highly Sensitive Pressure Sensors Based on Bionic Hierarchical Structures. Adv. Funct. Mater..

[2] Cao J., Lu C., Zhuang J., Liu M., Zhang X., Yu Y., Tao Q. (2017). Multiple Hydrogen Bonding Enables the Self-Healing of Sensors for Human-Machine Interactions. Angew. Chem. Int. Ed..

[3] Xiao Z., Zhou W., Zhang N., Zhang Q., Xia X., Gu X., Wang Y., Xie S. (2019). All-Carbon Pressure Sensors with High Performance and Excellent Chemical Resistance. Small.

[4] Chen X., Liu H., Zheng Y., Zhai Y., Liu X., Liu C., Mi L., Guo Z., Shen C. (2019). Highly Compressible and Robust Polyimide/Carbon Nanotube Composite Aerogel for High-Performance Wearable Pressure Sensor. ACS Appl. Mater. Interfaces.

[5] Shi H., Zhao X., Wu Z.-S., Dong Y., Lu P., Chen J., Ren W., Cheng H.-M., Bao X. (2019). Free-standing integrated cathode derived from 3D graphene/carbon nanotube aerogels serving as binder-free sulfur host and interlayer for ultrahigh volumetric-energy-density lithium sulfur batteries. Nano Energy.

[6] Wang X., Gu Y., Xiong Z., Cui Z., Zhang T. (2014). Silk-Molded Flexible, Ultrasensitive, and Highly Stable Electronic Skin for Monitoring Human Physiological Signals. Adv. Mater..

[7] Boland C.S., Khan U., Ryan G., Barwich S., Charifou R., Harvey A., Backes C., Li Z., Ferreira M.S., Möbius M.E. (2016). Sensitive electromechanical sensors using viscoelastic graphene-polymer nanocomposites. Science.

[8] Zhang F., Feng Y., Qin M., Gao L., Li Z., Zhao F., Zhang Z., Lv F., Feng W. (2019). Stress Controllability in Thermal and Electrical Conductivity of 3D Elastic Graphene-Crosslinked Carbon Nanotube Sponge/Polyimide Nanocomposite. Adv. Funct. Mater..

[9] Zhuo H., Hu Y., Tong X., Chen Z., Zhong L., Lai H., Liu L., Jing S., Liu Q., Liu C. (2018). A Supercompressible, Elastic, and Bendable Carbon Aerogel with Ultrasensitive Detection Limits for Compression Strain, Pressure, and Bending Angle. Adv. Mater..

[10] Song J., Guo X., Zhang J., Chen Y., Zhang C., Luo L., Wang F., Wang G. (2019). Rational design of free-standing 3D porous MXene/rGO hybrid aerogels as polysulfide reservoirs for high-energy lithium–sulfur batteries. J. Mater. Chem. A.

[11] Cai Y., Shen J., Ge G., Zhang Y., Jin W., Huang W., Shao J., Yang J., Dong X. (2018). Stretchable Ti_3_C_2_T_x_ MXene/Carbon Nanotube Composite Based Strain Sensor with Ultrahigh Sensitivity and Tunable Sensing Range. ACS Nano.

[12] An H., Habib T., Shah S., Gao H., Radovic M., Green M.J., Lutkenhaus J.L. (2018). Surface-agnostic highly stretchable and bendable conductive MXene multilayers. Sci. Adv..

[13] Jiang D., Zhang J., Qin S., Wang Z., Usman K.A.S., Hegh D., Liu J., Lei W., Razal J.M. (2021). Superelastic Ti3C2Tx MXene-Based Hybrid Aerogels for Compression-Resilient Devices. ACS Nano.

[14] Hu Y., Zhuo H., Chen Z., Wu K., Luo Q., Liu Q., Jing S., Liu C., Zhong L., Sun R.-C. (2018). Superelastic Carbon Aerogel with Ultrahigh and Wide-Range Linear Sensitivity. ACS Appl. Mater. Interfaces.

[15] Zhang J., Li B., Li L., Wang A. (2016). Ultralight, compressible and multifunctional carbon aerogels based on natural tubular cellulose. J. Mater. Chem. A.

[16] Li L., Li B., Sun H., Zhang J. (2017). Compressible and conductive carbon aerogels from waste paper with exceptional performance for oil/water separation. J. Mater. Chem. A.

[17] Li L., Hu T., Sun H., Zhang J., Wang A. (2017). Pressure-Sensitive and Conductive Carbon Aerogels from Poplars Catkins for Selective Oil Absorption and Oil/Water Separation. ACS Appl. Mater. Interfaces.

[18] Wu Z.-Y., Li C., Liang H.-W., Chen J., Yu S. (2013). Ultralight, Flexible, and Fire-Resistant Carbon Nanofiber Aerogels from Bacterial Cellulose. Angew. Chem..

[19] Wu X.-L., Wen T., Guo H.-L., Yang S., Wang X., Xu A.-W. (2013). Biomass-Derived Sponge-like Carbonaceous Hydrogels and Aerogels for Supercapacitors. ACS Nano.

[20] Wang C., Wu D., Wang H., Gao Z., Xu F., Jiang K. (2017). A green and scalable route to yield porous carbon sheets from biomass for supercapacitors with high capacity. J. Mater. Chem. A.

[21] Bi Z., Kong Q., Cao Y., Sun G., Su F., Wei X., Li X., Ahmad A., Xie L., Chen C.-M. (2019). Biomass-derived porous carbon materials with different dimensions for supercapacitor electrodes: A review. J. Mater. Chem. A.

[22] Jing Z., Ding J., Zhang T., Yang D., Qiu F., Chen Q., Xu J. (2019). Flexible, versatility and superhydrophobic biomass carbon aerogels derived from corn bracts for efficient oil/water separation. Food Bioprod. Process..

[23] Sam D.K., Sam E.K., Durairaj A., Lv X., Zhou Z., Liu J. (2020). Synthesis of biomass-based carbon aerogels in energy and sustainability. Carbohydr. Res..

[24] Hummers W.S., Offeman R.E. (1958). Preparation of Graphitic Oxide. J. Am. Chem. Soc..

[25] Marcano D.C., Kosynkin D.V., Berlin J.M., Sinitskii A., Sun Z., Slesarev A., Alemany L.B., Lu W., Tour J.M. (2010). Improved Synthesis of Graphene Oxide. ACS Nano.

[26] Stankovich S., Dikin D.A., Dommett G.H.B., Kohlhaas K.M., Zimney E.J., Stach E.A., Piner R.D., Nguyen S., Ruoff R.S. (2006). Graphene-based composite materials. Nat. Cell Biol..

[27] Zamiranvari A., Solati E., Dorranian D. (2017). Effect of CTAB concentration on the properties of graphene nanosheet produced by laser ablation. Opt. Laser Technol..

[28] Nawaz K., Khan U., Ul-Haq N., May P., O’Neill A., Coleman J.N. (2012). Observation of mechanical percolation in functionalized graphene oxide/elastomer composites. Carbon.

[29] Wang M., Shao C., Zhou S., Yang J., Xu F. (2018). Super-compressible, fatigue resistant and anisotropic carbon aerogels for piezoresistive sensors. Cellulose.

[30] Xu X., Zhou J., Nagaraju D.H., Jiang L., Marinov V.R., Lubineau G., Alshareef H.N., Oh M. (2015). Flexible, Highly Graphitized Carbon Aerogels Based on Bacterial Cellulose/Lignin: Catalyst-Free Synthesis and its Application in Energy Storage Devices. Adv. Funct. Mater..

[31] Cote L.J., Cruz-Silva R., Huang J. (2009). Flash Reduction and Patterning of Graphite Oxide and Its Polymer Composite. J. Am. Chem. Soc..

[32] Si Y., Yu J., Tang X., Ge J., Ding B. (2014). Ultralight nanofibre-assembled cellular aerogels with superelasticity and multifunctionality. Nat. Commun..

[33] Xu Y., Bai H., Lu G., Li C., Shi G. (2008). Flexible Graphene Films via the Filtration of Water-Soluble Noncovalent Functionalized Graphene Sheets. J. Am. Chem. Soc..

[34] Gholampour A., Kiamahalleh M.V., Tran D.N.H., Ozbakkaloglu T., Losic D. (2017). From Graphene Oxide to Reduced Graphene Oxide: Impact on the Physiochemical and Mechanical Properties of Graphene–Cement Composites. ACS Appl. Mater. Interfaces.

[35] Shen J., Hu Y., Shi M., Lu X., Qin C., Li C., Ye M. (2009). Fast and Facile Preparation of Graphene Oxide and Reduced Graphene Oxide Nanoplatelets. Chem. Mater..

[36] Qiu L., Liu J.Z., Chang S., Wu Y., Li D. (2012). Biomimetic superelastic graphene-based cellular monoliths. Nat. Commun..

[37] Jiang W., Yao C., Chen W., Li D., Zhong L., Liu C. (2020). A super-resilient and highly sensitive graphene oxide/cellulose-derived carbon aerogel. J. Mater. Chem. A.

[38] Si Y., Wang X., Yan C., Yang L., Yu J., Ding B. (2016). Ultralight Biomass-Derived Carbonaceous Nanofibrous Aerogels with Superelasticity and High Pressure-Sensitivity. Adv. Mater..

[39] Liang H.-W., Guan Q.-F., Chen L.-F., Zhu Z., Zhang W.-J., Yu S.-H. (2012). Macroscopic-Scale Template Synthesis of Robust Carbonaceous Nanofiber Hydrogels and Aerogels and Their Applications. Angew. Chem. Int. Ed..

[40] Xiao J., Tan Y., Song Y., Zheng Q. (2018). A flyweight and superelastic graphene aerogel as a high-capacity adsorbent and highly sensitive pressure sensor. J. Mater. Chem. A.

[41] Li C., Jiang D., Liang H., Huo B., Liu C., Yang W., Liu J. (2017). Superelastic and Arbitrary-Shaped Graphene Aerogels with Sacrificial Skeleton of Melamine Foam for Varied Applications. Adv. Funct. Mater..

