# Supplementary material for: Shape-Memory and Anisotropic Carbon Aerogel from Biomass and Graphene Oxide
Source: Molecules. 2021 Sep 21;26(18):5715. doi: 10.3390/molecules26185715 (PMC8464720; doi:10.3390/molecules26185715)
Supplement: Supplementary file 1 [file molecules-26-05715-s001.zip › molecules-1322224-supplementary.pdf]

molecules

Supplementary Material

Shape-Memory and Anisotropic Carbon Aerogel from Biomass and Graphene Oxide

Zilu Lin, Wenzhao Jiang, Zehong Chen, Linxin Zhong* and Chuanfu Liu*

State Key Laboratory of Pulp and Paper Engineering, South China University of Technology, Guangzhou 510641, China.
* Corresponding author. E-mail: lxzhong0611@scut.edu.cn (L. Z.); chfliu@scut.edu.cn (C. L.)

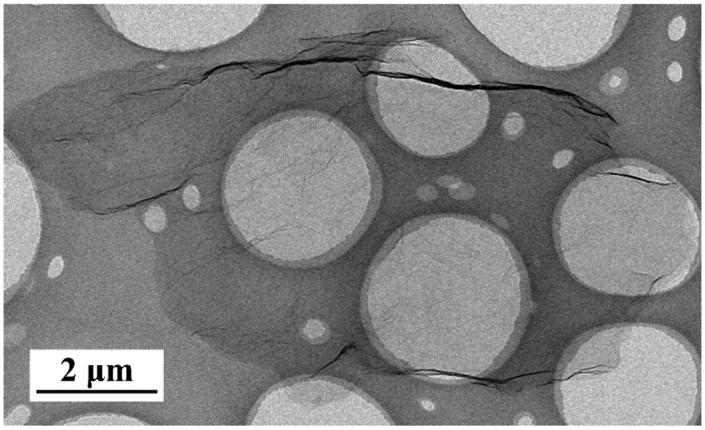

Figure S1. Transmission electron microscopy (TEM) image of GO.

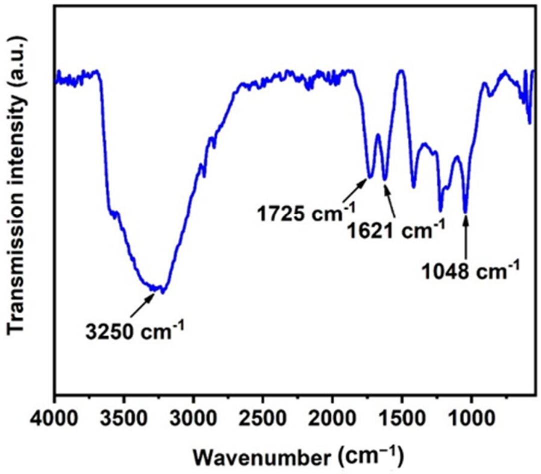

Figure S2. FTIR spectrum of GO.

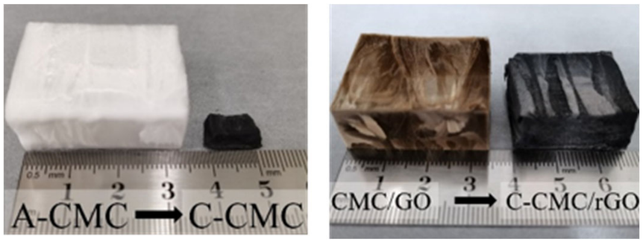

Figure S3. Digital photographs of CMC areogels and CMC/GO-4 (1.4 wt%) aerogels before and after annealing.

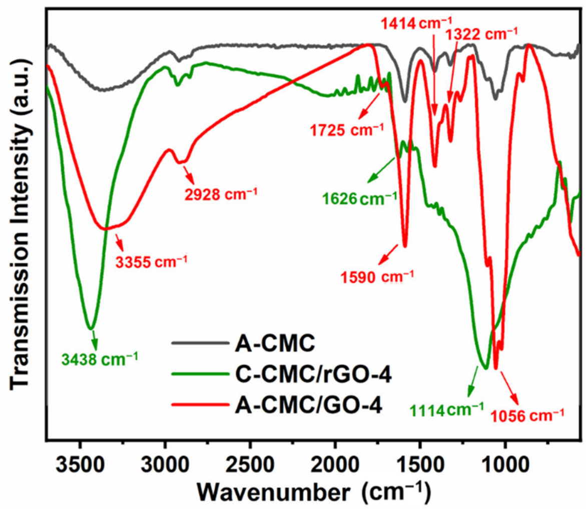

Figure S4. FTIR spectrua of A-CMC, A-CMC/GO-4 (1.4 wt%) and C-CMC/rGO-4.

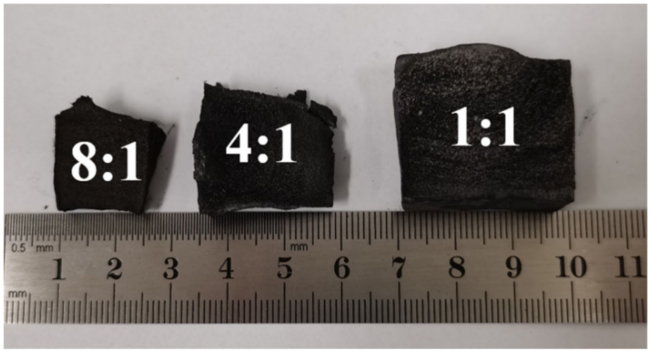

Figure S5. Digital photographs of C-CMC/rGO-1, C-CMC/rGO-4, C-CMC/rGO-8.

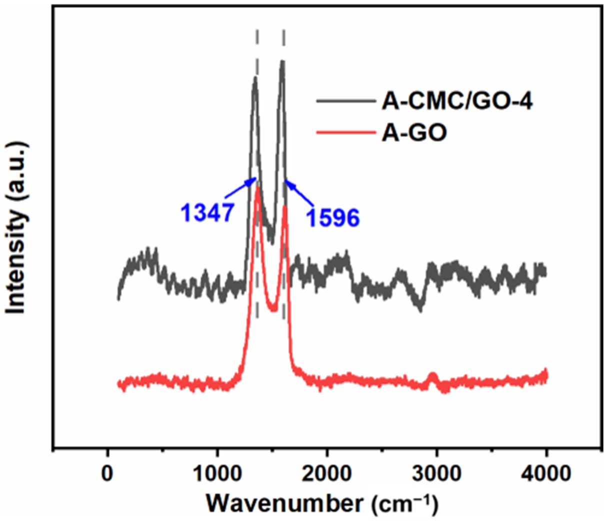

Figure S6. Raman patterns of A-CMC/rGO-4 and A-GO.

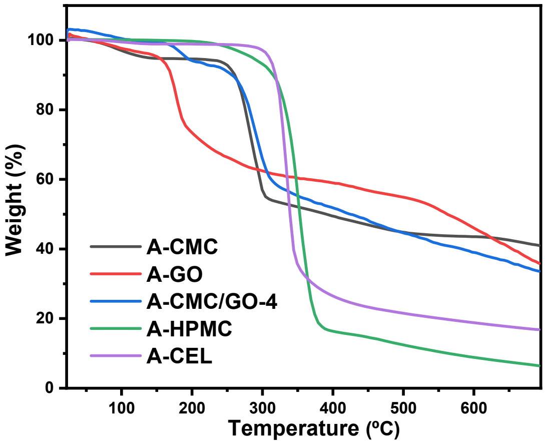

Figure S7. TGA curves of A-CMC, A-GO, A-HPMC, A-CEL, and A-CMC/GO-4.

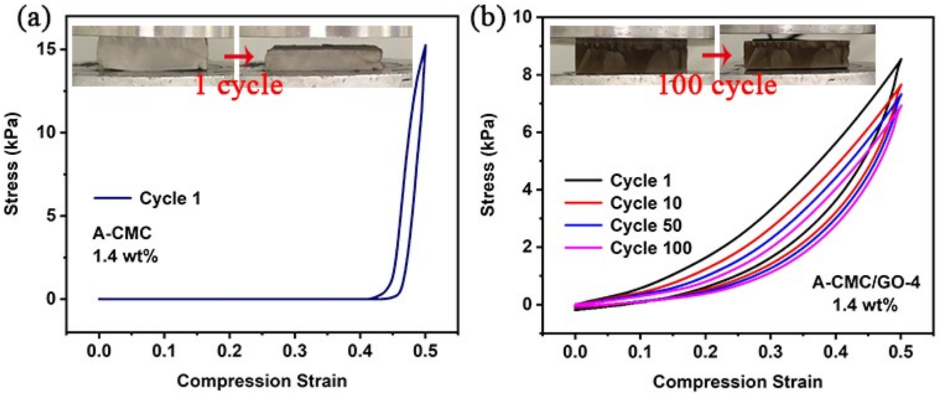

Figure S8. Stress-strain curves of (**a**) A-CMC and (**b**) A-CMC/GO-4.

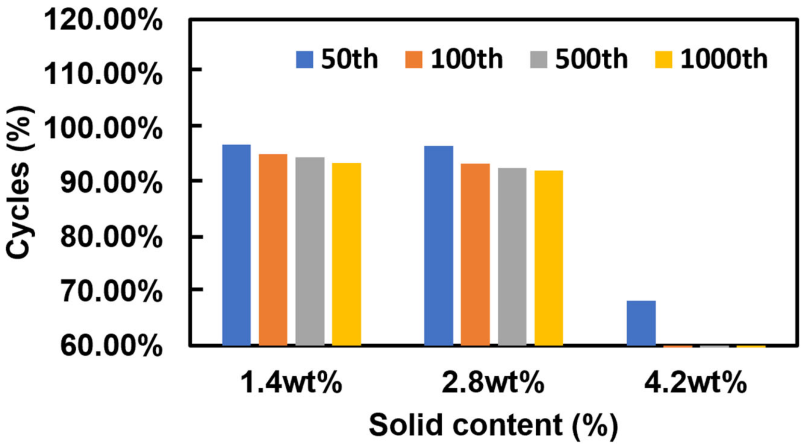

Figure S9. Stress retentions of C-CMC/rGO-4 with different solid contents at 50% strain.

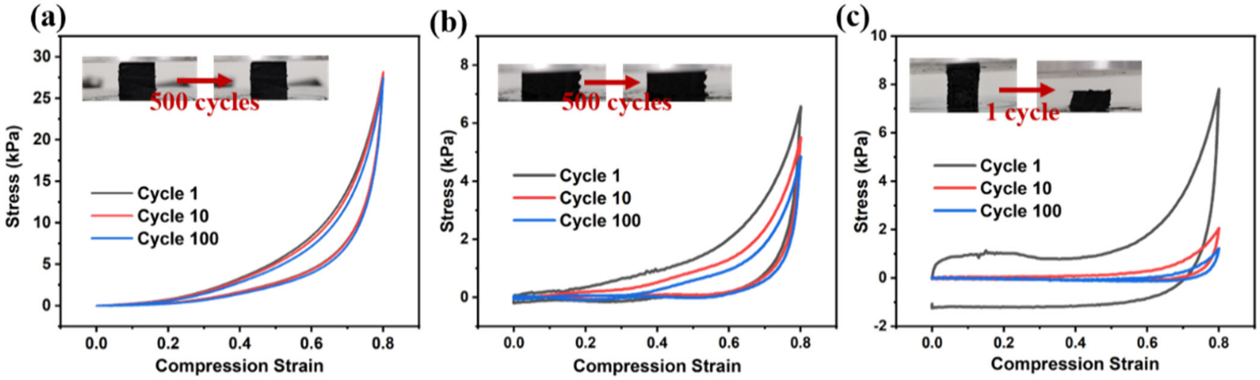

Figure S10. Stress-strain curves of C-CMC/rGO-4 from **(a)** the top, **(b)** lateral and, **(c)** the front.

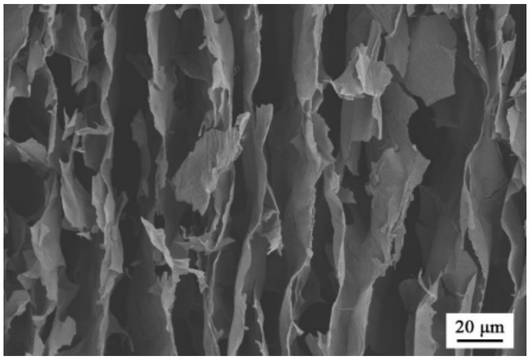

Figure S11. SEM image of C-CMC/rGO-4 with 4.2% solid content.

Table S1. BET surface area of the carbon aerogels.

Samples	C-CMC/rGO-1 (1.4 wt%)	C-CMC/rGO-4 (1.4 wt%)	C-CMC/rGO-8 (1.4 wt%)	C-CMC/rGO-1 (2.8 wt%)	C-CMC/rGO-1 (4.2 wt%)
S_{BET} (m^2/g)	35.7	72.9	179.3	54.4	79.6

Table S2. The comparison of stress retention of C-CMC/rGO-4 with those of other carbon aerogels.

Sample	Compression strain	Cycles	Stress retention rate
Ref.4	30%	1000	93.0%
Ref.35	50%	500	81.0%
Ref.36	50%	1000	76.0%
Ref.37	50%	1000	96.6%
	100%	300	76.6%
Ref.38	50%	1000	75.0%
Ref.39	60%	100	80.0%
Ref.40	60%	1000	72.0%
Ref.41	60%	1000	60.0%
Ref.42	90%	100	60.0%
This work	50%	50	97.0%
	50%	100	95.5%
	50%	500	94.7%
	50%	1000	93.6%
